# Supplementary material for: Effects of Isolated LAB on Chemical Composition, Fermentation Quality and Bacterial Community of Stipa grandis Silage
Source: Microorganisms. 2022 Dec 13;10(12):2463. doi: 10.3390/microorganisms10122463 (PMC9787380; doi:10.3390/microorganisms10122463)
Supplement: Supplementary file 1 [file microorganisms-10-02463-s001.zip › microorganisms-2066909-supplementary.pdf]

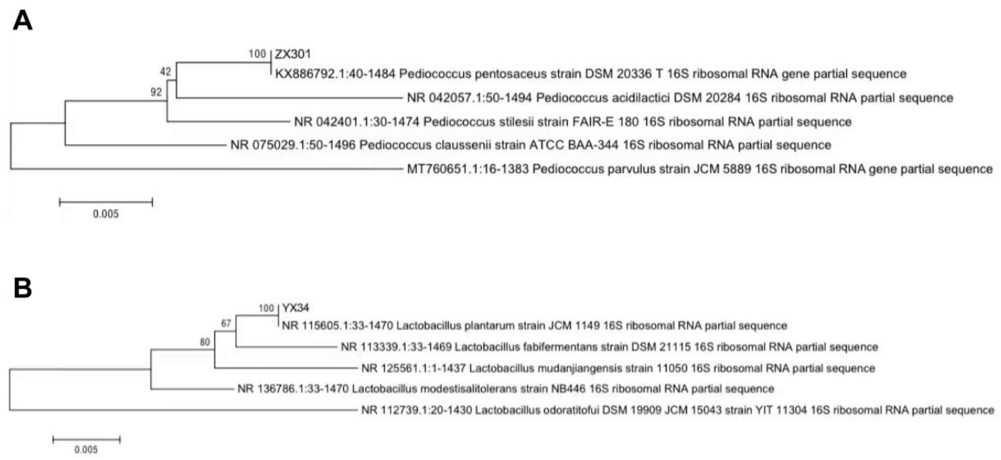

Figure S1. Phylogenetic tree of partial 16S rDNA sequences of isolated strains and sequences of identified bacteria in the nucleotide database of GenBank. (**A**) Phylogenetic tree of partial 16S rDNA sequences of ZX301 strains; (**B**) Phylogenetic tree of partial 16S rDNA sequences of YX34 strains.
